# SV-Pop: population-based structural variant analysis and visualization

**DOI:** 10.1186/s12859-019-2718-4

**Published:** 2019-03-13

**Authors:** Matt Ravenhall, Susana Campino, Taane G. Clark

**Affiliations:** 1 Department of Pathogen Molecular Biology, London School of Hygiene and Tropical Medicine, London, WC1E 7HT UK; 2 Department of Infectious Disease Epidemiology, London School of Hygiene and Tropical Medicine, London, WC1E 7HT UK

**Keywords:** Population genomics, Structural variation, Bioinformatics, Analytics, Python, R, Shiny

## Abstract

**Background:**

Genetic structural variation underpins a multitude of phenotypes, with significant implications for a range of biological outcomes. Despite their crucial role, structural variants (SVs) are often neglected and overshadowed by single nucleotide polymorphisms (SNPs), which are used in large-scale analysis such as genome-wide association and population genetic studies.

**Results:**

To facilitate the high-throughput analysis of structural variation we have developed an analytical pipeline and visualisation tool, called *SV-Pop*. The utility of this pipeline was then demonstrated through application with a large, multi-population *P. falciparum* dataset.

**Conclusions:**

Designed to facilitate downstream analysis and visualisation post-discovery, SV-Pop allows for straightforward integration of multi-population analysis, method and sample-based concordance metrics, and signals of selection.

## Background

Structural variation (SVs) describes changes to a core genome beyond single nucleotide polymorphisms (SNPs) or very short insertions and deletions (indels). Typically, SVs consist of four major types: deletions, insertions, duplications, and inversions. All play an important contribution to human and pathogen diversity and disease susceptibility. For example, duplications of the *Plasmodium falciparum* malaria parasite *gch1* have been associated with antimalarial resistance [1], and deletions of the human Duffy antigen convey resistance to malaria infection [2]. Despite their significant implications, the role of SVs has been overshadowed by SNPs, which can currently be identified easier and faster. Several SV discovery methods, such as *DELLY* and *CNVnator* currently exist [3, 4], but there is presently no tool for efficiently identifying concordance between models, up-scaling analysis for multiple populations, or visualising that output.

To assist the identification and investigation of SVs, we have developed a bioinformatics pipeline for high-throughput post-discovery analysis and visualisation that facilitates comparison across multiple populations and between different discovery methods.

### Implementation

**Fig. 1:**
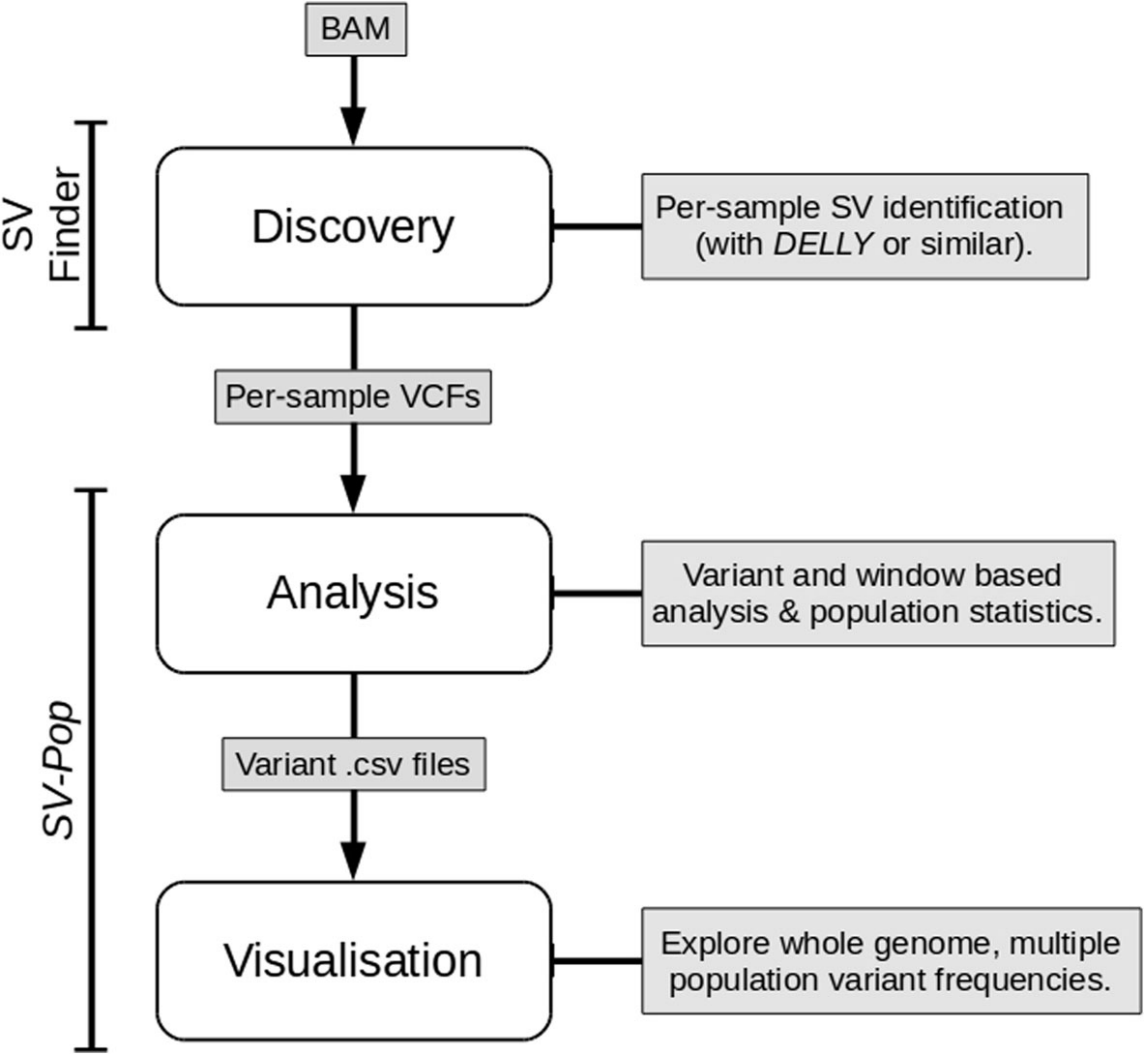
Summary of a typical *SV-Pop* run

*SV-Pop* consists of two core modules: (i) population-based analysis following individual SV discovery, and (ii) visualisation of those variants for dynamic, whole-genome exploration. The analysis module is a Unix command line tool built in Python (v3.3+) with *pandas* (v0.18+), and *numpy* (v1.10.4+). The visualisation module is built using the R Shiny web framework [5]⁠, and requires R (v3.3+) alongside the *shiny*, *plotly*, *data.table*, and *dplyr* packages. It can be launched on command line using ‘Rscript easyRun.r’, then explored via your default web browser. Input files should be pre-processed with *SV-Pop*, using the *PREPROCESS* mode for full compatibility. An overview of the full pipeline is shown in Fig. 1.

### Analysis

Input to *SV-Pop* consists of an array of post-discovery files (vcf format), one per-individual sample. These are typically the output of a run of *DELLY* or similar [3]. Variants across all samples are then processed, identifying and combining those specific variants that are shared across multiple samples and performing appropriate summary statistics. If so desired, variants can be filtered according to their concordance with a secondary discovery method by supplying a csv file of those variants with the *dirConcordance* argument. By default, variants are matched if they overlap at least 80% of the region identified by the primary method.

Once collated, we can consider a rolling window across the sample genome and identify regions with high or low variant overlap. This produces a coverage-like statistic for those underlying SVs. We can then further dissect according to sub-populations, as provided by the user. Specific variant sets can also be annotated, subset, merged, and filtered as required. In addition to core analysis and data processing functionalities, we have structured the pipeline to allow seamless integration of various filters and statistics, including method concordance and fixation indexes (F_ST_).

Typically, an analysis module run follows calling SVs across multiple models for a population of samples, inputting those individual output vcf files into *SV-Pop*, and producing per-variant or per-window based statistics (as csv files) for input into the visualisation module.

### Visualisation

Post-analysis, per-window files can be brought forward to the visualisation module, facilitating dynamic investigation of whole genome structural variation across multiple populations. By default, the visualisation module will identify variant frequencies and difference metrics (e.g. F_ST_ values) for all populations if present within your provided files, allowing the user to easily specify those they are interested in viewing. Similarly, the chromosomes and their sizes are detected allowing the user to specify regions of interest. Users are also able to subset and download specified genomic regions of interest for further analysis.

## Results

**Fig. 2:**
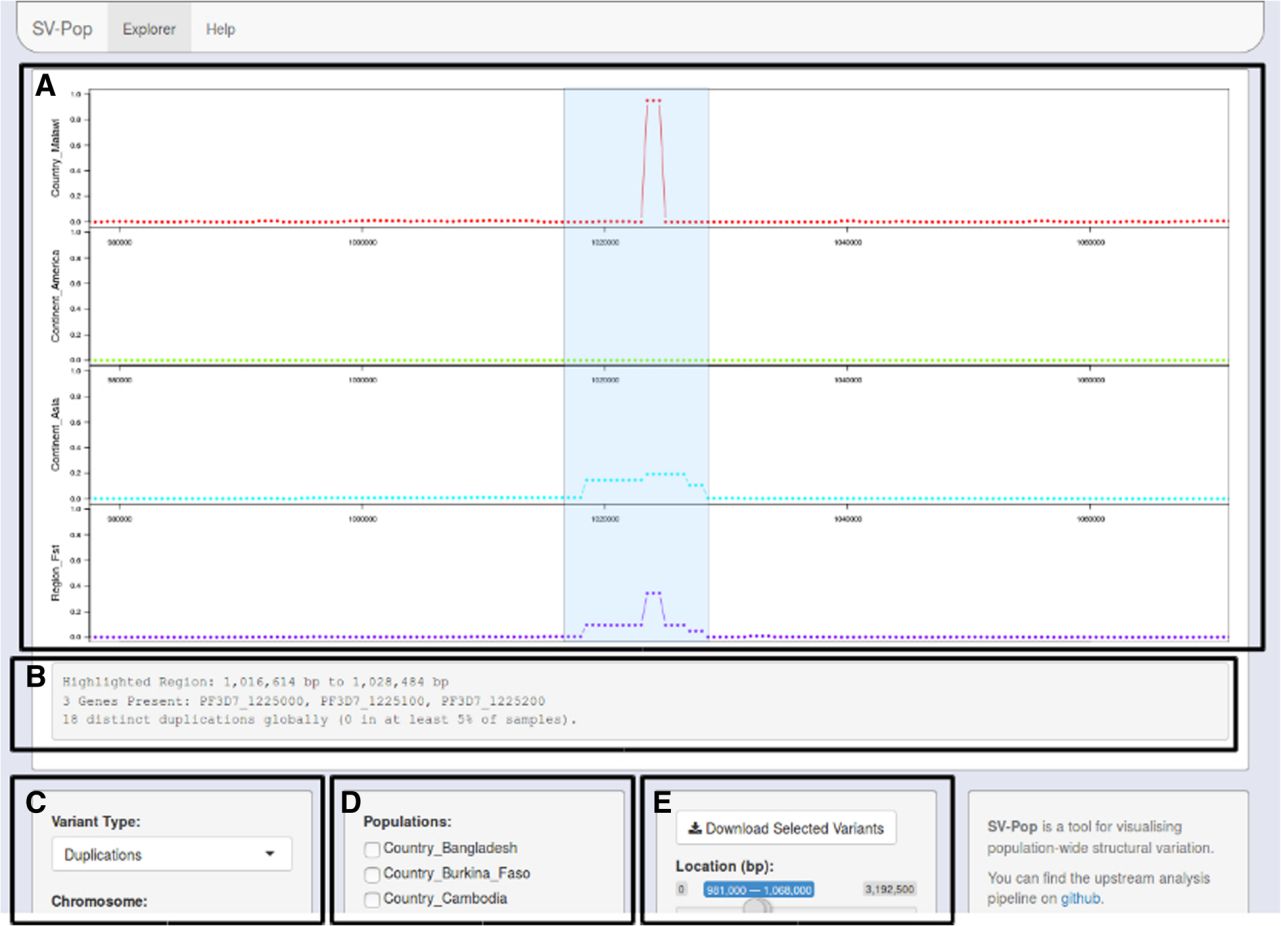
Screenshot of the visualization module displaying region-based F_ST_ values and window-based duplication frequencies for samples from Malawi, South America, and Asia. **a** Variant viewer, displaying per-window frequencies and statistical metrics. **b** Region summary, statistics regarding the region highlighted in the viewer. **c** Variant and Chromosome selector. **d** Population selection. **e** Location selection and download. The highlighted region demonstrated the presence of shorter three-window duplications in Malawi in contrast to an absence of duplications in South America and longer but less frequent duplications in Asia

To demonstrate the utility of *SV-Pop*, *P. falciparum* malaria parasite alignment files from 3110 samples across 21 countries with published sequence data [6]⁠ were processed with SV-Pop and loaded into the visualiser. As shown in Fig. 2, both elevated frequencies and a spike in the F_ST_ metric highlight the previously identified *gch1* promoter duplication.

The spike in the Malawi track (red) is the previously identified *gch1* promoter region duplication, whilst the ridge in the Asia track (cyan) indicates whole gene duplications. The F_ST_ track (purple) highlights frequency differences between region groups.

## Conclusions

*SV-Pop* dramatically increases the accessibility of large, population-based SV studies, allowing for a greater volume of downstream analysis and visualisation. It also establishes a core pipeline upon which to incorporate existing and future metrics such as method concordance and selection statistics. This implementation, which has been demonstrated on a *P. falciparum* dataset, is species-agnostic ensuring that it can be applied in a wide range of biological and geographical contexts.

## Availability and requirements

**Project name:** *SV-Pop.*

**Project home page:** https://github.com/mattravenhall/SV-Pop

**Operating system(s):** Unix (MacOS, Linux) or Windows 10.

**Programming language:** Python, R.

**Other requirements:** Python (3.3+): *numpy* (v1.10.4), *pandas* (v0.18); R (3.3+): *shiny*, *plotly*, *dplyr*, *data.table*. Included setup scripts will attempt to install all packages. Running on Windows 10 required use of the Bash shell.

**License:** MIT.

## Abbreviations

F_ST_: Fixation Index
SNP: Single Nucleotide Polymorphism
SV: Structural Variant
